# Comparison of Concordance of Peptic Ulcer Disease, Non-Adenomatous Intestinal Polyp, and Gallstone Disease in Korean Monozygotic and Dizygotic Twins: A Cross-Sectional Study

**DOI:** 10.3390/ijerph191912708

**Published:** 2022-10-04

**Authors:** Hyo Geun Choi, So Young Kim, Hyun Lim, Joo-Hee Kim, Ji Hee Kim, Seong-Jin Cho, Eun Sook Nam, Kyueng-Whan Min, Ha Young Park, Nan Young Kim, Sangkyoon Hong, Younghee Choi, Ho Suk Kang, Mi Jung Kwon

**Affiliations:** 1 Department of Otorhinolaryngology-Head & Neck Surgery, Hallym University Sacred Heart Hospital, Hallym University College of Medicine, Anyang 14068, Korea; 2 Department of Otorhinolaryngology-Head & Neck Surgery, CHA Bundang Medical Center, CHA University College of Medicine, Seongnam 13488, Korea; 3 Division of Gastroenterology, Department of Internal Medicine, Hallym University Sacred Heart Hospital, Hallym University College of Medicine, Anyang 14068, Korea; 4 Division of Pulmonary, Allergy, and Critical Care Medicine, Department of Medicine, Hallym University Sacred Heart Hospital, Hallym University College of Medicine, Anyang 14068, Korea; 5 Department of Neurosurgery, Hallym University Sacred Heart Hospital, Hallym University College of Medicine, Anyang 14068, Korea; 6 Department of Pathology, Kangdong Sacred Heart Hospital, Hallym University College of Medicine, Seoul 05355, Korea; 7 Department of Pathology, Hanyang University Guri Hospital, Hanyang University College of Medicine, Guri 11923, Korea; 8 Department of Pathology, Busan Paik Hospital, Inje University College of Medicine, Busan 47392, Korea; 9 Hallym Institute of Translational Genomics and Bioinformatics, Hallym University Medical Center, Anyang 14068, Korea; 10 Department of Pathology, Hallym University Dongtan Sacred Heart Hospital, Hallym University College of Medicine, Hwaseong 18450, Korea; 11 Research Insititute for Complementary & Alternative Medicine, Hallym University, Anyang 14068, Korea; 12 Department of Pathology, Hallym University Sacred Heart Hospital, Hallym University College of Medicine, Anyang 14068, Korea

**Keywords:** gastric ulcer, duodenal ulcer, intestinal polyp, cholelithiasis, cholangitis, monozygotic twins, dizygotic twins, environmental factor, genetic influence

## Abstract

Epidemiological studies have suggested the role of multiple genetic and environmental factors in the development of non-neoplastic gastrointestinal (GI) diseases; however, little information is available on these factors in the Korean population. Therefore, this cross-sectional study explored the effect of these factors by analyzing the concordance of several benign GI disorders in 525 monozygotic twins compared to that in 122 dizygotic twins aged >20 years from the Healthy Twin Study data of the Korean Genome and Epidemiology Study (2005–2014). Chi-square test, Wilcoxon rank-sum, and binomial and multinomial logistic regression models were used for statistical analysis. There was lack of concordance of gastric/duodenal ulcers and cholelithiasis/cholangitis between monozygotic twins compared to that in dizygotic twins, suggesting that environmental factors may mediate those concordant disease expressions in monozygotic twins. The concordance of intestinal polyps in monozygotic twins was 32% lower than that in dizygotic twins (*p* = 0.028), indicating that the effect of genetic factors on the risk for intestinal polyp development may be low. In conclusion, the lack or low concordance of several benign GI diseases between monozygotic and dizygotic twin groups suggests the relative importance of environmental factors, indicating that these are preventable diseases.

## 1. Introduction

Peptic ulcer disease (gastric and duodenal ulcer disease), intestinal non-adenomatous polyps, and cholelithiasis/cholangitis are common non-neoplastic gastrointestinal (GI) diseases in the adult population [1,2,3,4,5]. These diseases show considerable global variations in the prevalence and incidence in different ethnic populations, which suggests that the high occurrence of GI diseases in certain ethnic groups may be due to the presence of genetic factors involved in different metabolic pathways, combined with geographic environmental factors [6,7,8]. Epidemiological studies have proposed the interplay of multiple genetic and environmental elements in the development of non-neoplastic GI diseases, largely based on the European population [9,10,11,12,13,14]. However, little information is available regarding the relative importance of genetic and environmental contributions to these diseases in the Korean population. Korea is transitioning from being a developing country into a developed country with changing lifestyles and exogenous factors [15,16]. Therefore, it is necessary to evaluate the genetic and environmental contributions to common non-neoplastic GI diseases in Korea at this moment for identification of risk factors for disease prevention in the field of public health care.

Peptic ulcer disease is a condition characterized by painful sores caused by a defect in the protective layer of the inner lining of the stomach or duodenum resulting from secretion of gastric acid or pepsin. It potentially leads to serious complications such as GI bleeding, perforation, or gastric cancer if not treated promptly [17]. Previous reports have suggested an inherent influence in peptic ulcer disease based on a series of twin, family, and blood type studies in the European population [9,11,12,13,17]. Nevertheless, the effect of environmental predisposing factors such as the use of anti-inflammatory analgesics, smoking, alcohol consumption, illness, *Helicobacter pylori* infection, and mental stress has also been emphasized [1,10,18]. Given that Korea is one of the geographical regions with the highest prevalence of *Helicobacter pylori* infection [18,19] and the highest risk for gastric cancer worldwide [20,21,22], evaluation of the genetic and environmental influences on peptic ulcer disease in disease prevention and therapeutic management in Korea would provide valuable information.

Gallstone diseases, including cholelithiasis and cholangitis, are among the most common biliary tract disorders worldwide [5]. They are a major cause of surgery with considerable socioeconomic costs in most Western countries, with a high prevalence of 5.9–21.9% [5,23,24]. However, they have a much lower prevalence (2.2%) in Korea [25]. The possible risk factors for gallstone disease may be related to certain metabolic risk factors including fatty liver disease, increased age, obesity, and insulin resistance [25,26,27], whereas smoking and alcohol consumption seem to be controversial risk factors [14,25,28]. A meta-analysis indicated that differences in the prevalence among countries may be attributed to environmental factors such as living environments, lifestyle and dietary habits, smoking, physical activity, and culture [3]. To explain the discrepancy in the prevalence and risk factors between Korea and Western countries, it may be necessary to adjust for comprehensive confounding factors related to lifestyle.

An intestinal non-adenomatous polyp is a benign mass of tissue that arises from the bowel wall and protrudes into the lumen, including hyperplastic polyps, hamartomas, juvenile polyps, and pseudopolyps [2], accounting for approximately 50% of all types of intestinal polyps [29,30]. Most polyps do not cause symptoms, except for minor bleeding [2]. Despite having no malignant potential, they may grow more than 5 cm, and cause complications such as cramps, abdominal pain, or obstruction with a large lesion necessitating colonoscopic removal or surgical resection [31]. Although certain polyps, such as Peutz–Jeghers polyps, are linked with hereditary polyposis syndrome, sporadic cases of solitary Peutz–Jeghers-type polyps of the colon with unknown etiology have been reported [32]. The genetic and environmental factors that contribute to these non-adenomatous intestinal polyps are still unclear.

Twin studies may be useful in inferring the respective contribution of genetic and environmental factors to inter-individual variation in disease susceptibility [33]. In these studies, phenotypes of monozygotic and dizygotic twin pairs are compared. If concordance is observed to be significantly higher in monozygotic twins who share all their genes than in dizygotic twins who share half of their segregated genes, this is deemed an approximate indicator that genetic impacts are likely to be important [9]. Conversely, non-genetic and environmental factors are implicated if monozygotic twins are not fully concordant. Accordingly, this study aimed to evaluate the likelihood of genetic or environmental influence in a series of common benign GI diseases by comparing monozygotic twins (genetic influence) with dizygotic twin pairs (environmental contribution over genetic influence), while adjusting for comprehensive confounding factors.

## 2. Materials and Methods

### 2.1. Study Population and Data Collection

The study was conducted in accordance with the Declaration of Helsinki and was approved by the ethics committee of Hallym University (2021-03-004). Since the data used in the study was thoroughly anonymized, the requirement for written informed consent was waived by the Institutional Review Board.

This cohort study was based on the Healthy Twin Study (HTS), a nationwide cross-sectional survey that is a part of the prospective Korean Genome Epidemiology Study (KoGES, http://www.nih.go.kr/NIH/eng/main.jsp, accessed on 1 January 2020), that recruited Korean same-sex twin pairs aged over 20 years who primarily resided in Seoul or Busan, the two largest urban areas in Korea [34]. The participants were voluntarily recruited using advertisements and media at health-related governmental agencies and participating hospitals since 2005 [35,36,37]. The baseline data of KoGES HTS were obtained from 2005 to 2013 and follow-up data from 2008 to 2014. Zygosity was assessed at baseline using genetic analysis including 16 short tandem repeat markers (Amp*Fl*STR Identifier Kit; Perkin Elmer, Waltham, MA, USA) [38]. Two-thirds of the participants who completed the baseline examination were followed up, and their medical histories were updated. The study data were described in detail in previous studies [34,35,36,37].

### 2.2. Participants Selection

**Figure 1:**
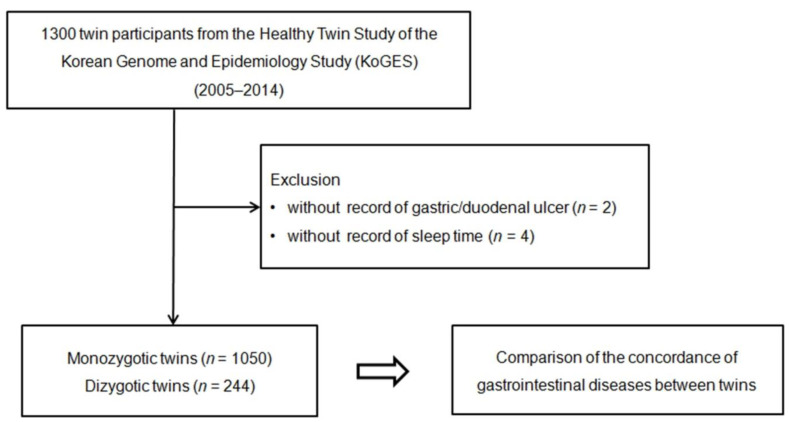
The study design of the present study. The 1050 monozygotic twins and 244 dizygotic twins were compared for the concordance of several common non-neoplastic gastrointestinal diseases including peptic ulcer disease, non-adenomatous intestinal polyp, and gallstone disease between twins.

A total of 1300 twin participants were recruited from the KoGES HTS database for this cross-sectional study. Participants without records of gastric/duodenal ulcers (n = 2) or sleep time (n = 4) were excluded from the study. Finally, 525 pairs of monozygotic (1050 participants) and 122 pairs of dizygotic twins (244 participants) were enrolled in the study (Figure 1). We then investigated the concordance of the history of non-neoplastic gastrointestinal diseases between the monozygotic and dizygotic twin groups.

### 2.3. Survey

Participants completed the interviewer-administered KoGES Baseline Core Questionnaire officially designed by the Korean National Institute of Health to collect information on demographic characteristics, lifestyle, including health conditions and medical history; the questionnaire is publicly available on the Korean National Institute of Health website (https://nih.go.kr/contents.es?mid=a50401010300) (accessed on 1 January 2022). Face-to-face interviews by trained interviewers were performed to clarify incomplete or ambiguous responses [34,39]. The diagnostically confirmed medical history of the participants was documented using a questionnaire including the history of non-neoplastic gastrointestinal diseases (gastric or duodenal ulcer, non-adenomatous intestinal polyp, cholelithiasis, or cholangitis). The listed questionnaires and data are regularly validated and updated. Interviewer-administered questionnaires, anthropometric measurements, and biochemical tests were conducted at the officially designated university hospitals and medical institutions, as described previously in detail [34].

Monthly income was categorized based on household income as non-respondent, low-income (<USD 2000 per month), middle income (USD 2000–3999 per month), and high income (≥USD 4000 per month). Educational status was classified as under high school, graduated from high school, dropped out of college, or graduated from college. Marital status was classified as unmarried, married, divorced, or otherwise. Physical activity levels were divided into hard and moderate, in addition to documenting walking time and sitting time in either the workplace or at home. Body mass index was expressed in kg/m^2^ using the health checkup data. Smoking history was classified as non-smoker (<100 cigarettes in entire life), past smoker (quit for more than 1 year), and current smoker. Drinking habits were grouped into non-drinkers, ≤1 time per month, 2–4 times per month, and ≥2 times per week. Sleep time was measured as 5/7 weekdays plus 2/7 weekends.

### 2.4. Exposure

Twin type (monozygotic and dizygotic) was regarded as an independent variable in the present study. Participants with multiple births, other than twins, were excluded.

### 2.5. Outcome

We estimated the concordance of the above-mentioned non-neoplastic several GI diseases between twin participants. These were sub-grouped as positive, positive-negative, or negative. If both twin siblings had (positive) or never had the same disease or trait (negative), they were considered concordant.

### 2.6. Statistical Analyses

Chi-square test (categorical variables) or Wilcoxon rank-sum test (continuous variables) was conducted to compare the baseline features of the participants. Odds ratios (ORs) with 95% confidence intervals (CIs) were calculated for the concordance of gastrointestinal diseases. First, the OR of monozygotic twins ((positive-positive or negative-negative)/(positive-negative)) was measured and compared with that of dizygotic twins using a binomial logistic regression model. Second, the OR of monozygotic twins ((positive-positive)/(positive-negative)/(negative-negative)/) was calculated and compared with that of dizygotic twins using a multinomial logistic regression model.

Crude, adjusted model 1 (sex, age, income, obesity, education, physical activity, frequency of alcohol consumption, smoking habit, marital status, sleep time, and medication history), and adjusted model 2 (model 1 plus the history of each disease (gastric/duodenal ulcer, intestinal polyp, and cholelithiasis/cholangitis)) were used to evaluate the findings. Two-tailed analyses were performed, and *p*-values less than 0.05 indicated significance. The results were statistically analyzed using SPSS (version 24.0; IBM, Armonk, NY, USA).

## 3. Results

Comparisons of the baseline features of monozygotic and dizygotic twins are summarized in Table 1. The rates of several benign GI diseases, including gastric/duodenal ulcers, non-adenomatous intestinal polyps, and cholelithiasis/cholangitis, did not significantly differ between monozygotic and dizygotic twins (all *p* > 0.05). The distribution of sex ratio, age groups, and hard physical activity level was different in monozygotic and dizygotic twins (*p* = 0.004, *p* = 0.016, and *p* = 0.013, respectively). Other variables such as education level, income level, other physical activity levels (except for the hard level), obesity, marital status, frequency of alcohol consumption, smoking status, and sleep duration were similar between the two groups (all *p* > 0.05).

**Figure 2:**
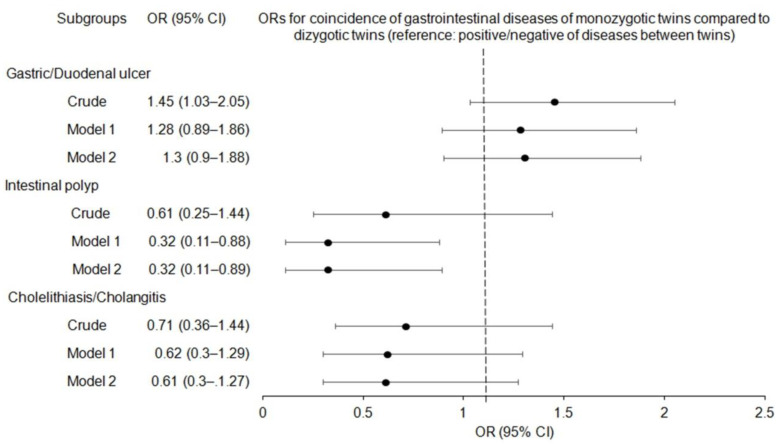
Forest plots for odds ratios (OR) (95% confidence intervals) (CI) of coincidence of gastric/duodenal ulcers, non-adenomatous intestinal polyp, cholelithiasis/cholangitis according to crude, model 1 (adjusting sex, age, income, obesity, education, physical activity, frequency of alcohol consumption, smoking habit, marital status, sleep time, and medication history), and model 2 (model 1 plus history of each disease (gastric/duodenal ulcer, intestinal polyp, and cholelithiasis/cholangitis)) analyses.

We investigated the concordance rates in terms of the presence or absence of gastric/duodenal ulcers, non-adenomatous intestinal polyps, and cholelithiasis/cholangitis in monozygotic twins compared to those in dizygotic twins (Table 2 and Figure 2). After adjusting for multiple covariates, the adjusted OR for the concordance rates of intestinal polyps was 32% lower in monozygotic twins than in dizygotic twins (0.32; 95% CI = 0.11–0.89; *p* = 0.028). Although the crude ORs for gastric/duodenal ulcers were significantly higher in the concordance rates of monozygotic twins (adjusted OR 1.45; 95% CI = 1.03–2.05; *p* = 0.034), the fully adjusted analyses revealed no statistically significant difference (1.30; 95% CI = 0.90–1.88; *p* = 0.169). The odds of concordance for cholelithiasis/cholangitis in monozygotic twins were not significantly higher than those in dizygotic twins (adjusted OR, 0.61; 95% CI = 0.30–1.27; *p* = 0.190).

**Figure 3:**
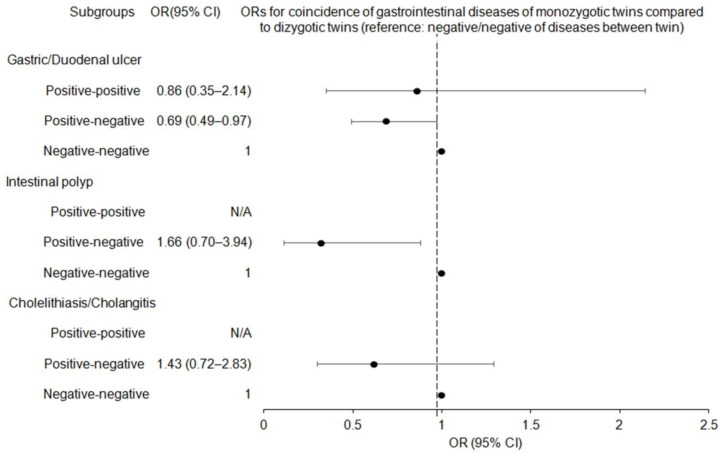
Forest plots for odds ratios (OR) (95% confidence intervals) (CI) of whether the incidence of gastric/duodenal ulcers, non-adenomatous intestinal polyp, and cholelithiasis/cholangitis was more common in monozygotic or dizygotic twins.

We further examined whether the incidence of several benign GI diseases was more common in monozygotic or dizygotic twins (Table 3 and Figure 3). In the crude and adjusted ORs, the incidence of gastric/duodenal ulcers, intestinal polyps, or cholelithiasis/cholangitis within twins was not significantly higher (all *p* > 0.05).

## 4. Discussion

A combined analysis of gastric/duodenal ulcers, non-adenomatous intestinal polyps, and cholelithiasis/cholangitis with full adjustments for lifestyle environmental factors has not been previously conducted in twin studies. Using validated adult Korean twin cohort data, this cross-sectional study indicates a lack of or low concordance of gastric/duodenal ulcers, non-adenomatous intestinal polyps, or cholelithiasis/cholangitis in monozygotic twins. We could not demonstrate any increased likelihood of several benign GI diseases in monozygotic or dizygotic twins after adjusting for multiple confounding factors and any mutual effects of these GI diseases. Because of the rarity of validated twin cohort data, our current twin study may further our understanding of the influence of environmental factors on the genetic contribution to gastric/duodenal ulcers, non-adenomatous intestinal polyps, or cholelithiasis/cholangitis in the Korean population.

The overall relationship between intestinal non-adenomatous polyps and twin pairs has rarely been determined. We noted that the concordance in the occurrence of non-adenomatous intestinal polyps exhibited a 32% lower likelihood in monozygotic twin pairs than in dizygotic twins; however, this finding requires careful interpretation. To evaluate the relative effect of genes to a trait, comparisons are applied between monozygotic and dizygotic twin concordance, with a higher monozygotic than dizygotic concordance rate entailing a role for genetics in estimating the trait [40]. Conversely, the greater likelihood of concordance in dizygotic twin pairs than in monozygotic twins sharing identical genes may indicate the relative importance of environmental influence on the risk for intestinal non-adenomatous polyp development. There is one case report of monozygotic twins with colorectal polyposis showing many hyperplastic polyps and adenomas. A genetic study revealed that the twins carried a rare missense variant of *BRCA2*. Furthermore, the number of intestinal polyps was significantly different between the twins and was positively correlated with smoking [41]. These observations in monozygotic twins suggest that both inherent and exogenous factors may be important in intestinal polyps.

In those with gastric/duodenal ulcer, concordance of the disease was associated with monozygotic twins in the crude model; this association lost significance after adjusting for sociodemographic and clinical factors including age, sex, obesity, smoking, alcohol, income, education, marriage status, physical activity, and sleep time. This analysis may suggest that environmental factors may mediate the concordant disease expression of gastric/duodenal ulcers in monozygotic twins. However, taken together with final adjusted analyses, the concordance or likelihood of the occurrence of gastric/duodenal ulcers and cholelithiasis/cholangitis was not higher in monozygotic twins than in dizygotic twins. The lack of concordance in the phenotypic presentation of gastric/duodenal ulcers and cholelithiasis/cholangitis within monozygotic twins may indicate the potential environmental effects relevant to dissimilar acquired lifestyle behaviors in monozygotic twins who share an identical genetic background, sex, and age. The discordance in phenotypic disease expression between monozygotic twin pairs may be partly explained by epigenetic differences [42], which may lead to the attenuation of inherited traits for disease occurrence via the regulation of gene expression by modifying chromatin accessibility to transcription factors [42]. For instance, genetically identical twins raised under different environmental circumstances have dissimilar lifespans or risks of developing GI diseases [40]. A recent twin study on peptic ulcer disease focused on the human gut microbiome composition has shown that aging and household-sharing may be the main determinants of gut microbial similarity and drift in both monozygotic and dizygotic twins [43]; however, there was a prominent decline in bacterial similitude with aging [43]. Another twin study indicated that histological gastric mucosa alterations showed high rates of concordance in both monozygotic and dizygotic twins [44], which suggests the importance of both genetic and environmental contributions in peptic ulcer disease. Likewise, in the present study, different environmental or acquired influences may have attenuated the chance of concordance of gastric/duodenal ulcers and cholelithiasis/cholangitis in monozygotic twin pairs compared to that in their dizygotic counterparts. Furthermore, because we included an adult population, environmental modifications may have altered the heritability of these diseases. Gene-environment interactions and epigenetic differences may result in discordance in disease expression between monozygotic twin pairs.

In the present study, although high concordance rates for gastric/duodenal ulcers (83.6% vs. 77.9%) and cholelithiasis/cholangitis (94.3% vs. 95.9%) were observed in monozygotic and dizygotic twins, no significant difference was observed between the two groups. Our results seem to be distinct from those of the European population-based twin study, in which the concordance rates of peptic ulcers were relatively low: 14.0% and 80.0% in monozygotic twins and 0% and 35.7% in dizygotic twins [10,45]. This may be explained by the much higher prevalence of gastric ulcer (3.3–3.4%) and duodenal ulcer (2.1–3.6%) in Korea, which may affect the concordance rates of peptic ulcer diseases within Korean twin pairs. In contrast, the global prevalence of peptic ulcer disease is 0.10–0.19% in outpatients and 0.03–0.17% in hospitalized patients [1].

Likewise, the lack of concordance of cholelithiasis/cholangitis in monozygotic twins contrasts with the Swedish twin study, which has reported higher concordances and correlations of cholelithiasis in monozygotic twins than in dizygotic twins [9]. Moreover, the concordance rates for cholelithiasis reported in previous studies in Finnish, Danish, and Swedish populations were 38% for monozygotic twins and 8% for dizygotic twins [9,46,47]. The discrepancy in our results may be partly due to ethnic differences. Furthermore, 20.8% of the Swedish twins with gallstone disease carried at least one *ABCG8* D19H allele, a gallstone candidate gene, which was linked with a 2.54-fold increased risk of gallstone disease [33]. However, the allele frequencies reported in the Taiwanese population are different from those reported in the Swedish study as the *ABCG8* D19H allele is quite rare (1.4%) [48].

The strength of the current study is the use of prospective twin cohort data with follow-up for both monozygotic and dizygotic twins from the KoGES HTS. The data was of high quality and was subjected to regular validation by national statisticians, which made our findings more reliable. We comprehensively considered the potential confounders among lifestyle factors, including smoking, obesity, physical activity, sleep duration, and alcohol drinking, as well as socioeconomic factors, including income level, education level, and marital status for comparisons between twin pairs. Adjustments for the aforementioned lifestyle factors may be further strengthen the study because these factors have been documented as possible risk factors for gastric/duodenal ulcers, non-adenomatous intestinal polyps, or cholelithiasis/cholangitis [2,8,10,26,28]. To the best of our knowledge, no large-scale twin studies have been conducted on gastric/duodenal ulcers, non-adenomatous intestinal polyps, or cholelithiasis/cholangitis in the Korean population.

This study had several limitations that should be addressed. First, although a considerable number of variables were adjusted for in the present study, unmeasured confounders could not be completely excluded. Second, although validated questionnaires were applied to examine the medical histories of GI diseases that previously had been diagnostically confirmed in the medical institutions, there can be recall bias due to the retrospective interview of the participants. Third, the causal relationship between twin births and benign GI diseases could not be confirmed by the cross-sectional study design. Fourth, the limited number of twin pairs with gastric/duodenal ulcers, intestinal non-adenomatous polyps, or cholelithiasis/cholangitis may limit the generalizability of the results, although the study represents one of the largest samples of twin participants. Finally, the lack of data on *Helicobacter pylori* or genetic data on related gastric/duodenal ulcers, intestinal polyps, or cholelithiasis/cholangitis may be an additional limitation.

## 5. Conclusions

In summary, this study indicates a lack of concordance of gastric/duodenal ulcers and cholelithiasis/cholangitis and much lower likelihood of concordance of non-adenomatous intestinal polyps in monozygotic twins compared to that in dizygotic twin groups in the Korean population. Our data may highlight the potential importance of preventable environmental factors for several incidents that are common but economically burdensome GI diseases. This study further highlights the importance of personal health care and prevention in patients with these GI diseases.

## Figures and Tables

**Table 1:** The baseline features of the monozygotic and dizygotic twins.

Characteristics		Total Participants		
		Monozygotic Twin	Dizygotic Twin	*p*-Value
Age (years old, n, %)				0.004 *
	20–24	6 (0.6)	0 (0)	
	25–29	68 (6.5)	4 (1.6)	
	30–34	362 (34.5)	87 (35.7)	
	35–39	244 (23.2)	65 (26.6)	
	40–44	139 (13.2)	36 (14.8)	
	45–49	129 (12.3)	20 (8.2)	
	50–54	82 (7.8)	22 (9)	
	55–59	14 (1.3)	10 (4.1)	
	60–64	4 (0.4)	0 (0)	
	65+	2 (0.2)	0 (0)	
Sex (n, %)				0.016 *
	Males	384 (36.6)	134 (54.9)	
	Females	666 (63.4)	244 (100)	
Income (n, %)				0.978
	<2 million (KRW)	349 (33.2)	81 (33.2)	
	2 to <3 million (KRW)	282 (26.9)	68 (27.9)	
	3 to <4 million (KRW)	214 (20.4)	50 (20.5)	
	≥4 million (KRW)	205 (19.5)	45 (18.4)	
Education (n, %)				0.743
	Under high school	122 (11.6)	25 (10.2)	
	Graduated from high school	371 (35.3)	92 (37.7)	
	Commercial college/dropped out of college	123 (11.7)	32 (13.1)	
	Graduated from high school	434 (41.3)	95 (38.9)	
Marriage (n, %)				0.302
	Unmarried	240 (23.1)	50 (20.5)	
	Married	733 (70.5)	173 (70.9)	
	Divorced or others	67 (6.4)	21 (8.6)	
Physical Activity				
	Hard (hour/week, mean, SD)	3.1 (6.8)	4.7 (9.7)	0.013 *
	Moderate (hour/week, mean, SD)	5.8 (10.5)	6.2 (10.2)	0.612
	Walk (hour/week, mean, SD)	6.1 (9.6)	6.8 (10.9)	0.291
	Sit (hour/week, mean, SD)	40.1 (22)	37.9 (20.7)	0.155
Obesity (n, %)				0.203
	Underweight (BMI < 18.5)	27 (2.6)	5 (2)	
	Normal (BMI ≥ 18.5 to < 23)	510 (48.6)	113 (46.3)	
	Overweight (BMI 23 to < 25)	220 (21)	68 (27.9)	
	Obese I (BMI ≥ 25 to < 30)	261 (24.9)	52 (21.3)	
	Obese II (BMI ≥ 30)	32 (3)	6 (2.5)	
Smoking status (n, %)				0.138
	Nonsmoker	691 (65.8)	145 (59.4)	
	Past smoker	108 (10.3)	33 (13.5)	
	Current smoker	251 (23.9)	66 (27)	
Frequency of drinking alcohol (n, %)				0.314
	Nondrinker	304 (29)	64 (26.2)	
	≤1 time per month	238 (22.7)	46 (18.9)	
	2–4 times per month	300 (28.6)	80 (32.8)	
	≥2 times per week	208 (19.8)	54 (22.1)	
Sleeping hours (n, %)				0.370
	≤5 h	53 (5)	16 (6.6)	
	6–7 h	619 (59)	146 (59.8)	
	8–9 h	350 (33.3)	72 (29.5)	
	≥10 h	28 (2.7)	10 (4.1)	
Gastrointestinal diseases (categorical)				
	Gastric/duodenal ulcer (n, %)	111 (10.6)	32 (13.1)	0.254
	Intestinal polyp (n, %)	23 (2.2)	3 (1.2)	0.451
	Cholelithiasis/cholangitis (n, %)	35 (3.3)	5 (2)	0.319

* Significance at *p* < 0.05. Chi-square test (categorical variables) or Wilcoxon rank-sum test (continuous variables) was used.

**Table 2:** Analysis of odds ratios with 95% confidence intervals of concordance of gastrointestinal diseases of monozygotic twins compared to those of dizygotic twins (reference: positive/negative of diseases between twin).

Concordance of Diseases	Monozygotic Twin	Dizygotic Twin	Odds Ratios (95% Confidence Interval)					
	n (%)	n (%)	Crude	*p*	Model 1 †	*p*	Model 2 ‡	*p*
Gastric/duodenal ulcer								
concordant	878/1050 (83.6)	190/244 (77.9)	1.45 (1.03–2.05)	0.034 *	1.28 (0.89–1.86)	0.187	1.30 (0.90–1.88)	0.169
discordant	172/1050 (16.4)	54/244 (22.1)	1		1		1	
Intestinal polyp								
concordant	1008/1050 (96.0)	238/244 (97.5)	0.61 (0.25–1.44)	0.256	0.32 (0.11–0.88)	0.028 *	0.32 (0.11–0.89)	0.028 *
discordant	42/1050 (4.0)	6/244 (2.5)	1		1		1	
Cholelithiasis/cholangitis								
concordant	990/1050 (94.3)	234/244 (95.9)	0.71 (0.36–1.44)	0.317	0.62 (0.30–1.29)	0.204	0.61 (0.30–1.27)	0.190
discordant	60/1050 (5.7)	10/244 (4.1)	1		1		1	

* Significance at *p* < 0.05. † Adjusted for age, sex, income, education, marriage status, physical activity, obesity, smoking habit, frequency of drinking alcohol, and sleep time. ‡ Model 1 plus histories of each disease (gastric/duodenal ulcer, intestinal polyp, and cholelithiasis/cholangitis). “Concordant” means concordant positive-positive or negative-negative result between monozygotic twins or between dizygotic twins, whereas “discordant” means discordant positive and negative results between monozygotic twins or between dizygotic twins.

**Table 3:** Analysis of odds ratios with 95% confidence intervals of concordance of gastrointestinal diseases of monozygotic twins compared to those of dizygotic twins (reference: negative/negative of diseases between twin).

Concordance of Diseases	Monozygotic Twin	Dizygotic Twin	Odds Ratios (95% CI)					
	n (%)	n (%)	Crude	*p*	Model 1 *	*p*	Model 2 †	*p*
Gastric/duodenal ulcer								
Positive-positive	24/1050 (2.3)	6/244 (2.5)	0.86 (0.35–2.14)	0.748	1.01 (0.39–2.63)	0.990	1.00 (0.38–2.61)	0.994
Positive-negative	172/1050 (16.4)	54/244 (22.1)	0.69 (0.49–0.97)	0.032 *	0.73 (0.51–1.05)	0.088	0.72 (0.51–1.04)	0.077
Negative-negative	854/1050 (81.3)	184/244 (75.4)	1		1		1	
Intestinal polyp								
Positive-positive	2/1050 (0.2)	0/244 (0.0)	N/A	N/A	N/A	N/A	N/A	N/A
Positive-negative	42/1050 (4.0)	6/244 (2.5)	1.66 (0.70–3.94)	0.254	2.04 (0.82–5.07)	0.125	2.05 (0.82–5.11)	0.124
Negative-negative	1006/1050 (95.8)	238/244 (97.5)	1		1		1	
Cholelithiasis/cholangitis								
Positive-positive	6/1050 (0.6)	0/244 (0.0)	N/A	N/A	N/A	N/A	N/A	N/A
Positive-negative	60/1050 (5.7)	10/244 (4.1)	1.43 (0.72–2.83)	0.309	1.57 (0.78–3.16)	0.209	1.58 (0.78–3.19)	0.201
Negative-negative	984/1050 (93.7)	234/244 (95.9)	1		1		1	

* Adjusted for age, sex, income, education, marriage status, physical activity, obesity, smoking habit, frequency of drinking alcohol, and sleep time. † Model 1 plus histories of each disease (gastric/duodenal ulcer, intestinal polyp, and cholelithiasis/cholangitis).

## Data Availability

Restrictions apply to the availability of these data. Data were obtained from the Korean Genome and Epidemiology Study (KoGES) and are available at https://www.nih.go.kr/contents.es?mid=a50401010100#1 (accessed on 1 January 2022).
